# Takotsubo Cardiomyopathy in a Polytrauma Patient With Subarachnoid Hemorrhage

**DOI:** 10.7759/cureus.38954

**Published:** 2023-05-12

**Authors:** Mithun Murali Krishna, Mrinal Murali Krishna, Meghna Joseph

**Affiliations:** 1 Emergency Medicine, Pushpagiri Institute of Medical Sciences & Research Centre, Thiruvalla, IND; 2 Internal Medicine, Government Medical College, Thiruvananthapuram, Thiruvananthapuram, IND; 3 Pediatrics, Government Medical College, Thiruvananthapuram, Thiruvananthapuram, IND

**Keywords:** traumatic brain injury, pulmonary edema, subarachnoid hemorrhage, neurogenic stunned myocardium, takotsubo cardiomyopathy

## Abstract

Takotsubo cardiomyopathy (TCM) is a sudden, transient myocardial stunning precipitated by severe emotional or physical stress. It is characterized by left ventricular apical ballooning and elevated cardiac enzymes without significant coronary artery stenosis. Stress-induced catecholamine surge has been proposed to be the likely mechanism of TCM. We report the case of a 23-year-old female who presented to the emergency department unconscious and in respiratory distress following a motor vehicle accident. The point-of-care ultrasonography showed prominent B lines in bilateral lung fields and a dilated inferior vena cava (IVC). An x-ray and computed tomography (CT) scan of the chest revealed bilateral diffuse ground glass opacities. A CT scan of the brain showed a subarachnoid hemorrhage (SAH). Electrocardiography (ECG) showed normal sinus rhythm, but troponin I was elevated. Echocardiography revealed left ventricular apical hypokinesia. The coronary angiogram was normal. A diagnosis of TCM with SAH was made. Appropriate emergent care was provided, and at follow-up, she made a complete cardiologic recovery. TCM is a puzzling condition in an emergency setting and accurate and timely diagnosis is imperative in the management. Early prevention of hypoxemia and maintenance of mean arterial pressure and cerebral perfusion pressure is critical in determining the long-term outcome of the patient in the setting of co-existing CNS pathologies.

## Introduction

Takotsubo cardiomyopathy (TCM) is a sudden, transient myocardial stunning characterized by apical ballooning of the left ventricle (LV) with no significant coronary artery stenosis. It was first described by Sato et al. in Japan in 1990. Patients often present with chest pain, elevated cardiac enzymes, and electrocardiography (ECG) changes typical of an acute coronary syndrome (ACS). The syndrome is often precipitated by severe emotional or physical stress [1]. 

The diagnosis of TCM can be made using the modified Mayo Clinic criteria, which require the presence of regional wall motion abnormalities (RWMAs) of the left ventricle extending beyond a single epicardial vessel distribution, the absence of coronary artery obstruction or acute plaque rupture in angiography, new ECG abnormalities or elevation of cardiac enzymes, and the absence of myocarditis or pheochromocytoma [2].

The exact pathophysiology of TCM still needs to be completely understood. Stress-induced catecholamine surge has been proposed to be the likely reason for TCM [3]. Microvascular abnormalities [4] or direct myocardial toxicity caused by neurohormonal stimulation are considered to cause acute myocardial dysfunction. Central nervous system (CNS) disorders have also been found to trigger TCM [5]. Traumatic brain injury may lead to TCM or neurogenic stunned myocardium (NSM), in which the patient may rapidly progress into acute left ventricular failure and cardiogenic shock. Women are found to be more affected by this condition [6].

Hypotension and cardiogenic pulmonary edema are the most common immediate complications of TCM due to acute LV dysfunction. Dysrhythmias, mitral regurgitation, LV thrombus formation, LV rupture, and death can also occur in the acute setting [7]. TCM is managed symptomatically, and the syndrome has an excellent prognosis with complete cardiovascular recovery in most patients within weeks [8]. This case emphasizes the importance of keeping TCM as a differential diagnosis in a trauma patient presenting with unexplained shock.

## Case presentation

**Figure 1:**
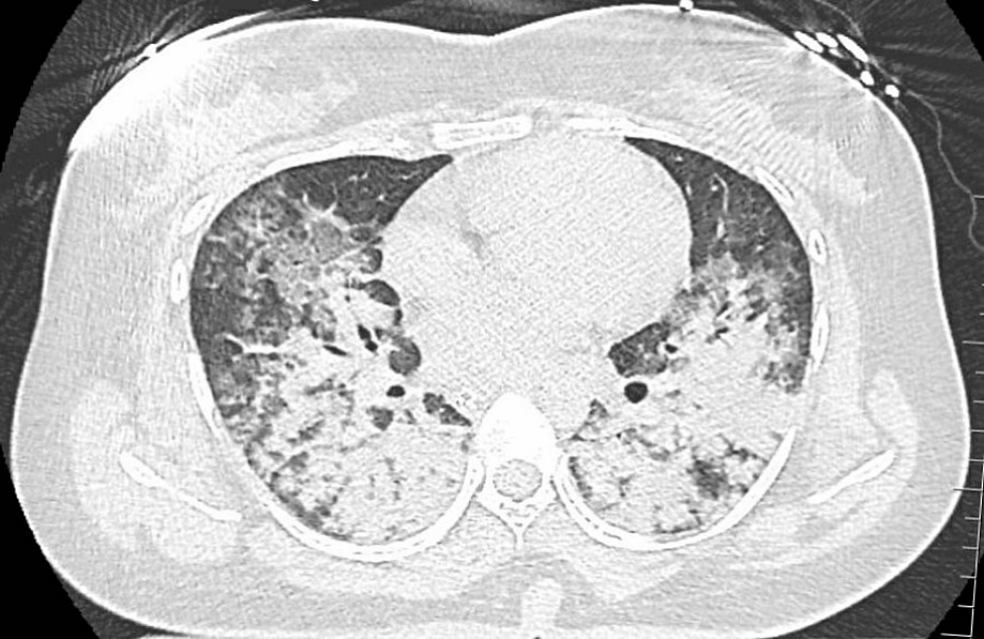
CT chest. CT chest showing bilateral pulmonary consolidation and ground glassing. CT: computed tomography.

**Figure 2:**
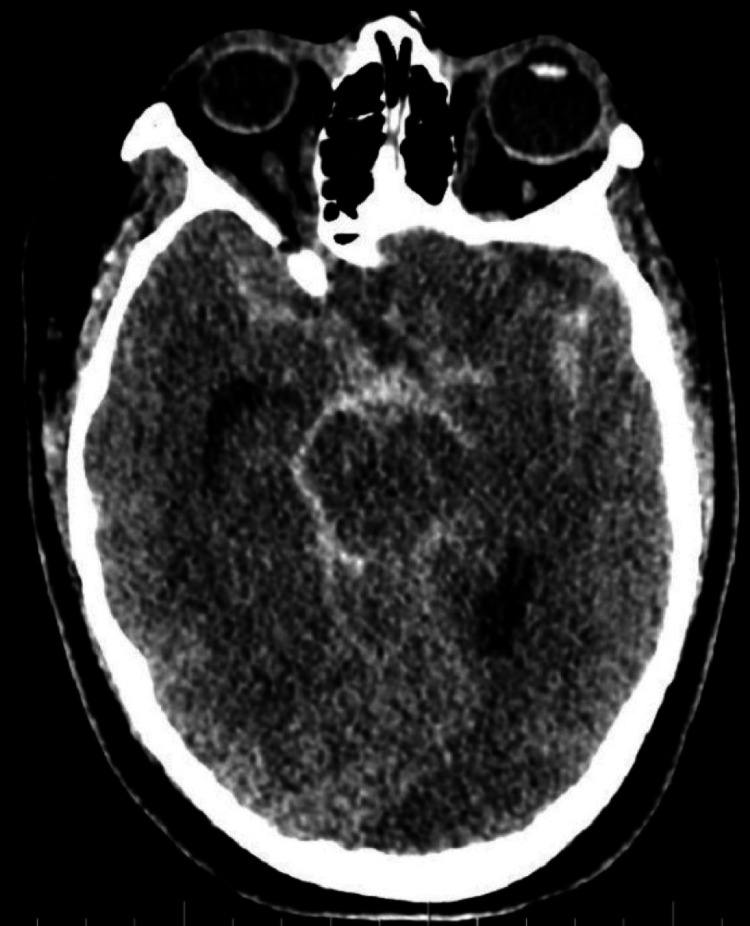
CT brain. CT brain showing subarachnoid hemorrhage. CT: computed tomography.

A 23-year-old female presented to the emergency department (ED) with a history of a motor vehicle accident. On arrival in the ED, the patient was in an altered sensorium with a Glasgow Coma Scale (GCS) score of E2V3M5. She was in respiratory distress, with a respiratory rate of 36/minute and an oxygen saturation of 75% on room air. Her heart rate was 126 beats per minute, and her blood pressure (BP) was initially 100/70 mmHg. Two large-bore intravenous cannulas were secured, and intravenous fluids were initiated. The patient was intubated with an 8.0 endotracheal tube, fixed at 20 centimeters (cm) from the incisors, and put on mechanical ventilation in volume-controlled ventilation (VCV) mode. Her saturation improved to 90%. Pink froth was coming through the endotracheal tube, suggestive of pulmonary edema. Extended focused assessment with sonography in trauma (eFAST) study showed no free fluid in pleural, pericardial, or peritoneal cavities, but there were prominent B lines in bilateral lung fields in all four zones of both lungs. The inferior vena cava (IVC) measured 2.1 centimeters in diameter with no variation in respiration (Video 1). She was assessed with a chest x-ray, which showed bilateral diffuse infiltrates, again suggestive of pulmonary edema. The ECG showed a normal sinus rhythm. Echocardiography (Video 2) showed LV apical hypokinesia and basal hyperkinesia. Her fluids were stopped in view of pulmonary edema. Soon she developed hypotension, and her BP dropped to 70/50 mmHg. A noradrenaline infusion was initiated, which was titrated to maintain a BP of 120/70 mmHg. She was administered three doses of furosemide (40 milligrams) injections intravenously and continued on noradrenaline. The troponin I (TnI) level was 444 ng/mL. After hours of continued treatment, the oxygen saturation improved to 94% on mechanical ventilation set at a fraction of inspired oxygen (FiO_2_) of 60%. Computed tomography (CT) of the chest (Figure 1) showed multiple discrete confluent areas of consolidation and ground glassing in both lungs. A CT scan of the brain (Figure 2) showed subarachnoid hemorrhage (SAH). Repeat TnI was 3080 ng/mL. A cardiology evaluation was sought. She was admitted to the intensive care unit. Coronary angiography was done and was found to be normal. A diagnosis of TCM/NSM with SAH was made. Antiepileptics, antibiotics, and antifibrinolytics were given for SAH management. She was maintained on noradrenaline support, weaned off, and extubated after two days when her GCS score improved to 14/15. Her BP was maintained without any pressor support. She was started on ivabradine and carvedilol. She was shifted to the neurology intensive care unit for further management. At follow-up after six weeks, she made a complete cardiologic recovery with no residual cardiac dysfunction.

**Video 1:** eFAST. eFAST showing plethoric IVC not collapsing with respiration. eFAST: Extended focused assessment with sonography in trauma, IVC: inferior vena cava.

**Video 2:** Echocardiography. Echocardiography showing LV apical hypokinesia and basal hyperkinesia. LV: left ventricle.

## Discussion

In a patient presenting to the ED with altered sensorium, hypotension, and respiratory distress following polytrauma, the differential diagnoses include conditions like pneumothorax, hemothorax, cardiac contusion, aortic dissection, or other aortic injuries, pericardial effusion, pulmonary contusion, acute coronary syndrome (ACS), or cardiogenic pulmonary edema [9]. A thorough trauma evaluation is warranted for these patients. Internal or external hemorrhage should be ruled out in a patient presenting with severe hypotension following polytrauma. eFAST and chest x-ray help rule out pulmonary, pleural, pericardial, or aortic pathologies.

**Figure 3:**
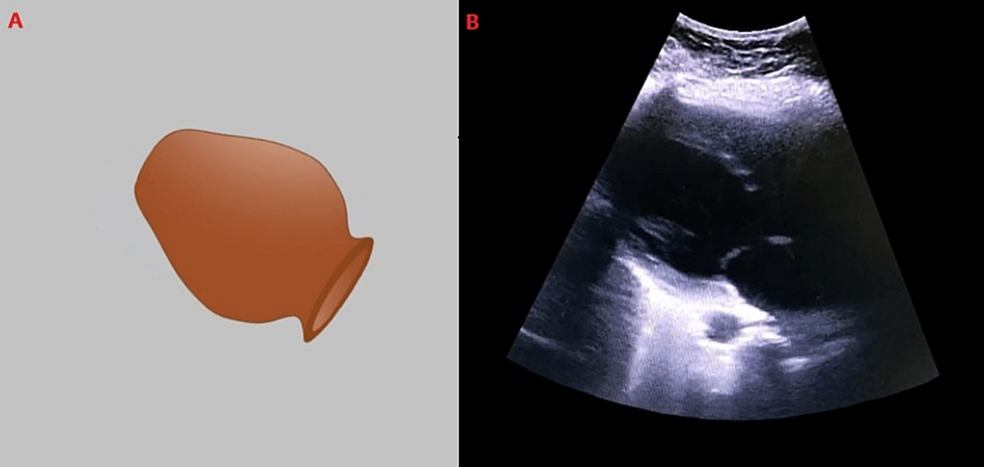
Comparison of the appearances of a Japanese octopus pot and the left ventricle in TCM. Panel (A) shows a Japanese octopus pot. Panel (B) shows the characteristic appearance of the left ventricle in TCM due to apical ballooning. TCM: Takotsubo cardiomyopathy.

Hypotension unresponsive to intravenous fluid therapy and worsening pulmonary edema point to a cardiac pathology as the cause for the acute clinical presentation. An echocardiogram showed LV apical ballooning and hypokinesia with basal hyperkinesia, typical of TCM. The appearance of the heart is often compared to that of a Japanese octopus pot (Figure 3). Specific ECG changes can occur in TCM, though none were observed in this patient [10]. Though very unlikely in a young patient, additional evaluation for possible ACS was done with coronary angiography due to elevated plasma troponin levels and transthoracic echocardiography (TTE) findings. The left ventricular wall motion abnormalities, characteristic of TCM can be detected with a bedside TTE in the ED. Such changes can also follow ACS, hence the importance of the evaluation of coronary patency. The InterTAK Diagnostic Score can be used to differentiate TCM from ACS in the acute setting [11].

TCM generally presents as an acute cardiovascular compromise characterized by severe left ventricular dysfunction. TCM, however, differs from an ACS because patients generally have a normal coronary angiogram. The left ventricular dysfunction extending beyond the territory subtended by a single coronary artery recovers within a few days or weeks [12]. It is usually associated with acute emotional or physical stress, but cases have also been reported with no identifiable triggers. The precise pathophysiology of TCM is yet to be deciphered, but catecholamine-induced myocardial stunning is the most accepted explanation. An excess of catecholamines can lead to significant myocardial structural alterations, which in turn impair the microcirculation and cause direct cardiotoxicity [13]. 

TCM is one of the most frequently encountered cardiac debilities precipitated by CNS disorders like subarachnoid hemorrhage [14]. SAH can lead to a neurogenic stunned myocardium due to an increase in local catecholamine levels mediated by neuronally transmitted norepinephrine [15]. Experiments have shown that in the acute phase of subarachnoid hemorrhage, elevated sympathetic nervous system activity can induce myocardial damage and consequent dysfunction [16]. The patient in this case has SAH, which could have contributed to the development of TCM. The myocardium affected in TCM has good potential for structural reconstitution, which explains the rapid functional recovery seen in this patient [13].

The apical ballooning in TCM can be explained by the regional differences in the density of β-adrenoreceptors in the heart. β2-adrenoceptors are more frequently expressed in the apical than in the basal segments of the LV, whereas β1-adrenoceptors are expressed much more at the base than at the apex of the LV [17]. Supraphysiological levels of epinephrine can trigger β2-adrenoceptors in the apex to switch from Gs to Gi coupling [18], causing a negative inotropic effect resulting in apical hypokinesia. This is a protective mechanism aimed at preventing cardiotoxicity from the simultaneous activation of β1 and β2 adrenoceptors during situations of catecholamine excess [19].

The clinical course of TCM is typically self-limiting, with complete recovery within weeks [1]. Emergent management predominantly includes the judicious use of intravenous fluids and vasopressor support for hypotension. Intra-aortic balloon pumping can be given to patients with vasopressor-resistant hypotension. As myocardial stunning is transient in TCM, hemodynamic derangement is oftentimes brief and reversible. Anticoagulants are warranted in patients with LV thrombus. Dysrhythmias in the acute setting, a major determinant of patient outcome, should also be managed appropriately [20]. Cardiology follow-up is recommended for patients to confirm the resolution of the cardiomyopathy and for long-term management.

## Conclusions

TCM is a puzzling condition in an emergency setting, and physicians might fall short of identifying this unique diagnosis, especially in this age group. An accurate and timely diagnosis is imperative, as the management is substantially different from other possible differential diagnoses. Unwarranted use of fluids and blood products to correct hypotension in a patient with NSM caused by trauma can be catastrophic. Maintenance of mean arterial pressure to prevent hypoxemia and to maintain cerebral perfusion pressure is challenging and critical in TCM/NSM in coexisting CNS pathologies like SAH. Other alternative intrathoracic diagnoses like pneumothorax, cardiac contusion, aortic injury, pericardial effusion, pulmonary contusion, acute coronary syndrome, or other primary cardiac pathologies causing pulmonary edema must be ruled out.
